# Erosive rhinitis resembling granulomatosis with polyangiitis (Wegener's granulomatosis) in an Anatolian shepherd dog

**DOI:** 10.4102/jsava.v86i1.1187

**Published:** 2015-04-21

**Authors:** Marlies Böhm, Sandra Basson

**Affiliations:** 1 King Edward Veterinary Referral Hospital, Port Elizabeth, South Africa; 2 Drs Visser, Erasmus, Vawda & Partners, Port Elizabeth, South Africa

## Abstract

Granulomatosis with polyangiitis (Wegener's granulomatosis) is one of the idiopathic immune-mediated small-vessel vasculitides described in humans which are characterised by the presence of circulating antineutrophil cytoplasmic antibodies. It most commonly involves capillaries, venules and arterioles of the ear, nose and throat, lungs and glomeruli. A case of destructive haemopurulent rhinitis associated with relapsing periods of pyrexia, lethargy and stiffness as well as generalised pulmonary infiltrates in a young Anatolian shepherd dog is presented that closely resembles granulomatosis with polyangiitis (GPA) as reported in humans. Perinuclear antineutrophil cytoplasmic antibodies (pANCA) were detected in the dog's serum. Signs resolved promptly and completely once immunosuppressive doses of prednisone were administered, and have not recurred. This is the first report on the use of pANCA to investigate rhinitis in dogs. It is also, to the authors’ knowledge, the first description of a relapsing haemopurulent lytic rhinitis in this species. The concurrent manifestations of erosive haemopurulent rhinitis, ground-glass opacities on pulmonary computed tomography, pyrexia and listlessness resemble GPA as described in humans.

## Introduction

Wegener's granulomatosis was first described by Dr Friedrich Wegener in 1936 (Jennette 2011; Santana et al. 2011). In 2011 its name was changed to granulomatosis with polyangiitis (GPA) (Falk et al. 2011). Ear, nose and throat signs that include a destructive rhinitis that may erode nasal cartilages andor nasal bones, serous otitis media, gingivitis and oral ulcers are the most common presenting signs and affect 85% – 95% of patients. Pulmonary haemorrhages and/or nodules are evident in 40% – 60% and glomerulonephritis develops in 40% – 70% of cases (Pagnoux & Wolter 2012). Most affected people have lesions in several organs at the time of diagnosis, and typically have systemic signs including fever, weight loss, arthralgia and myalgia. Characteristic neutrophil-rich granulomas centring on arterioles, venules and capillaries are only evident on biopsy in a minority of cases (Devaney et al. 1990; Jennette 2011; Pagnoux & Wolter 2012).

Diagnosis is typically made based on algorithms that include characteristic clinical signs; changes in serum biochemistry, urine analysis and diagnostic imaging, histopathology and the presence of antineutrophil cytoplasmic antibodies (ANCA) in serum samples (Ozaki 2007) are used once infectious diseases and malignancies have been excluded (Pagnoux & Wolter 2012; Watts et al. 2007). It is thought that ANCA play a central role in the pathogenesis of this condition (Kallenberg 2010).

## Case history

**FIGURE 1:**
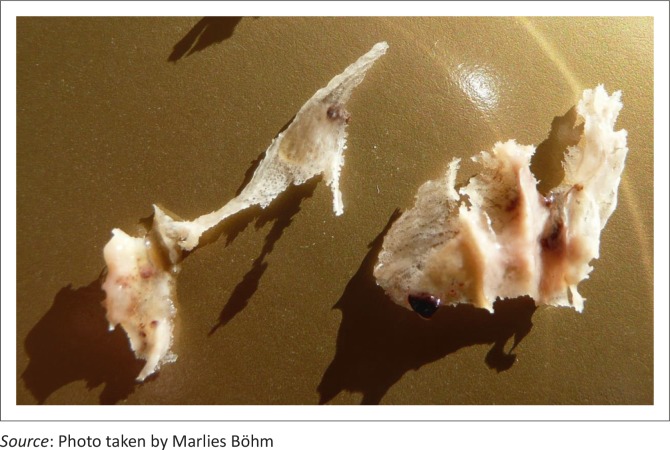
Necrotic nasal turbinates sneezed out by the patient during the second episode.

An 8-month-old female entire Anatolian shepherd dog developed four episodes of pyrexia that lasted 3–8 days and were associated with generalised stiffness, lethargy and rhinitis. Nasal discharge was initially serous, turned haemorrhagic and finally became purulent. During the haemorrhagic phases blood clots were sneezed out. During the purulent phase of the second episode she sneezed out necrotic nasal turbinates (Figure 1). The dog appeared clinically normal during the 3–6 weeks between periods of pyrexia. Treatment with parenteral prednisolone for 2 days, clavulanate potentiated amoxycillin, amoxycillin and doxycycline (doses and manufacturers not recorded) had not improved signs by the time she was referred, 6 days into the first episode.

Clinical examination revealed the following additional abnormalities during each episode: rectal temperature initially fluctuating between 40.4 °C and 41.1 °C (normal 38.0 °C – 39.0 °C) and then normalising over 1–2 days, mild generalised lymphadenopathy and mild facial pain. Although the dog appeared stiff, the following were not detected: focal muscle pain on palpation, neck pain, muscle wasting, palpable joint effusion, neurological deficits. Retinal examination was unremarkable, there was no nasal stertor and nasal airflow appeared symmetrical and normal.

During each episode the dog became neutropenic and developed a left shift (mature neutrophils 2.46 x 10^9^/L and bands of 1.61 x 10^9^/L during the first episode; normal range for mature neutrophils is 3–11.8 x 10^9^/L; for bands < 0.3 x 10^9^/L). Neutrophils showed mild toxic changes. Haematocrit was low-normal [0.373 L/L (normal 0.37–0.55)] during the first episode, but well within the reference range from the end of episode three until 2 days into episode four (0.438 L/L – 0.524 L/L). There were no parasites on a capillary blood smear.

A smear of the nasal discharge during a neutropenic episode revealed large numbers of neutrophils with rare extracellular bacteria. This suggested that the periods of neutropenia developed as a consequence of increased peripheral demand rather than decreased neutrophil production. Seven blood samples collected at 2–7-day intervals between the third and fourth episodes showed neutrophil numbers within the normal range at all times, including on day 2 of the fourth episode. This eliminated a primary cyclic neutropenia as the cause of the pyrexia.

Platelet count, prothrombin time and partial thromboplastin time were normal during the first episode. The systolic blood pressure was 125 mmHg (UltraTec PD1v, Ultrasound Technologies Ltd, Caldicot, UK; cuff size 5). Urine specific gravity was greater than 1.040 (normal > 1.030) and dipstick analysis revealed a trace of protein with an inactive sediment; therefore the urine protein creatinine ratio was not determined. In view of the normal urine analysis, a urine culture was not performed. Serum biochemistry (proteins, liver enzymes, urea and creatinine, potassium and calcium levels) showed no significant abnormalities. Thus epistaxis developed as a result of local rather than systemic disease. Blood cultures were not performed. Faecal flotation and direct smears revealed no pathogens.

**FIGURE 2:**
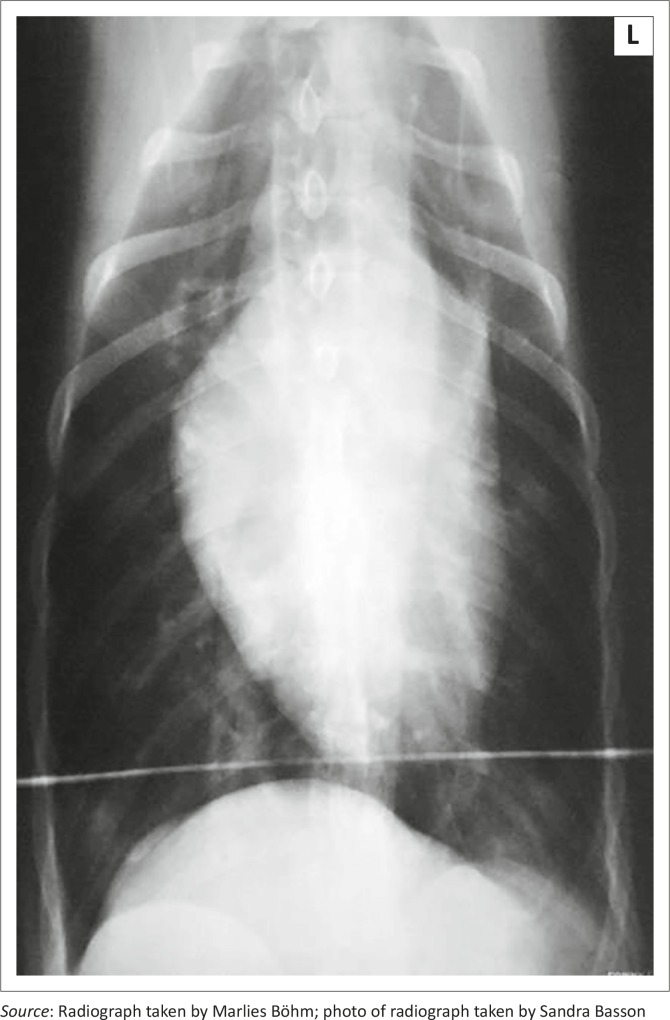
Ventrodorsal thoracic radiograph. The radiodense horizontal line caudal to the heart is the edge of the cradle the dog was lying in to assist positioning. Note subtle diffuse increase in radiodensity at the edges of the caudal lung lobes.

**FIGURE 3:**
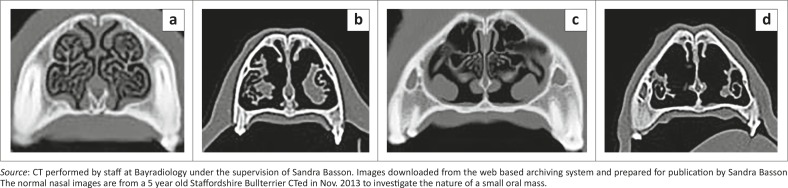
Nasal CT images: (a) rostral nasal chambers in a normal dog, and (b) in the patient showing some turbinate beading and destruction of the finer cartilage scrolls W 3077, C570; (c) middle nasal chambers in a normal dog and (d) in the patient showing severe turbinate lysis/atrophy W 3077, C 570.

**FIGURE 4:**
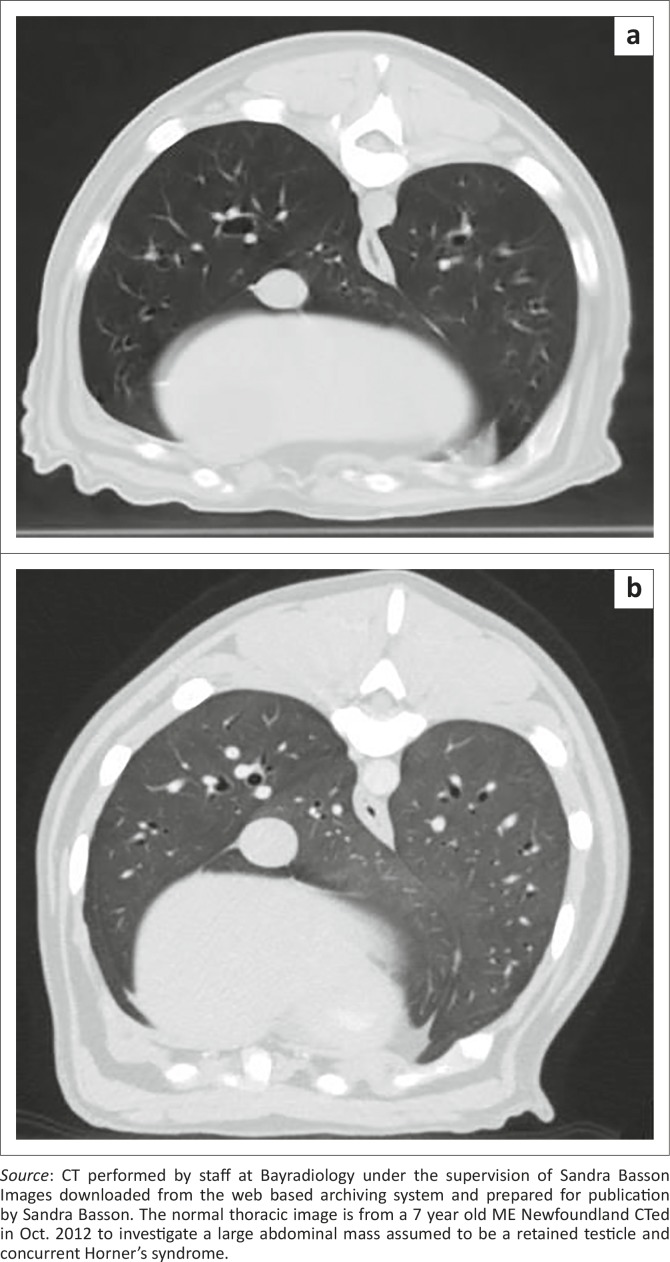
(a) Normal canine lungs and (b) ground-glass appearance of patient’s lungs W 1465 C -498.

Thoracic radiography during the first episode revealed a peripheral interstitial pattern that was most severe in the caudodorsal lung lobes (Figure 2). Echocardiography was unremarkable (standard B- and M-mode views and measurements, as well as colour flow Doppler using a Philips HDI 5000 with 5 Mhz – 8 Mhz microconvex and 3 MHz – 5MHz phased array probes). During the second episode 0.625 mm helical computed tomography (CT) (Lightspeed RT16, General Electric Company, Milwaukee, USA) images of the head and thorax were acquired before as well as after intravenous administration of 15 mL Imeron 400 (Bracco Imaging S.p.A., Millan, Italy). The CT revealed a dramatic, bilateral lytic rhinitis and sclerosing osteitis that affected the middle nasal chambers most severely (Figure 3). The mucosa overlying the turbinates appeared thickened in the rostral third of the nose, especially on the right. Ethmoturbinates appeared beaded (i.e. there were multifocal areas of thickened mucosa consistent with granulomata), but there were no significant changes in the sinuses. The lungs had a generalised ground-glass appearance (Figure 4).

Endoscopy was performed once on the 7th day of the second episode, using an Olympus GIF XP20 fibre-optic gastroscope (7.9 mm insertion tube) and an Akcn xenon light source for the bronchoscopy and posterior rhinoscopy, and switching to a Karl Storz 2.8 mm rigid 30° arthroscope with a sheath for the anterior rhinoscopy. The nasal cavity was flushed with saline under pressure through the sheath whilst performing anterior rhinoscopy. Nasal decongestants were not used. The tracheal and bronchial mucosa appeared normal. No discharge or haemorrhage was evident. Posterior rhinoscopy was unremarkable. On anterior rhinoscopy the turbinates in the rostral third of the nose appeared normal. In the middle third the mucosa appeared hyperaemic and cobblestoned. The turbinates were atrophied with large cavities evident bilaterally. Lesions were bilateral but subjectively worse in the right nasal chambers. On biopsy the turbinates felt soft. Broncho-alveolar lavage and airway brushings were not collected for reasons of cost: the imaging findings and physical appearance led us to believe that such samples were highly unlikely to yield a diagnosis.

Histopathology revealed thick, fibrinocellular material composed of neutrophils, fibrin, mucous and sloughed epithelial cells adherent to nasal mucosa. There were many exocytosing inflammatory cells in the respiratory epithelium. Dense proprial infiltrates of lymphocytes and plasma cells were evident, with prominent stromal oedema. Necrosis of turbinate bone was noted in some sections. In one such area large clustered colonies of Gram-positive cocci and Gram-negative rods were encountered. Periodic acid-Schiff stain revealed no fungi. There were rare early possible granulomas. On review the pathologist felt that although there were changes that were consistent with those described for Wegener's granulomatosis in man, they were not diagnostic.

*Escherichia coli* and *Enterococcus* were cultured from nasal biopsies collected during the second episode. Both organisms were sensitive to clavulanate potentiated amoxicillin, despite the fact that the dog had been treated with this antibiotic (Synulox, Pfizer Animal Health, Isando, South Africa; 17.4 mg/kg twice daily) during her second neutropenic phase, with the last treatment 5 days prior to the biopsy. Fungal and mycoplasma cultures were negative. Polymerase chain reaction (PCR) was unable to detect herpes virus or *Bartonella* in the biopsies.

Serum was submitted to the Clinical Investigations Centre of the Royal Veterinary College in London. Perinuclear antineutrophil cytoplasmic antibodies (pANCA) were detected by immunofluorescent assay, as previously described (Allenspach et al. 2004).

Treatment with prednisone (Be-Tabs Prednisone, Be-Tabs Pharmaceuticals, Industria, South Africa) at 1 mg/kg twice daily was begun on day 2 of the fourth episode. Pyrexia, the stiff and stilted gait and the haemorrhagic nasal discharge resolved within 24 hours. The dose was slowly tapered over 5 months and then discontinued. Signs have not recurred in the 30 months since the start of the treatment.

## Discussion

This case is unusual for three reasons: disease manifestation was cyclical; severe lytic rhinitis was associated with pyrexia, neutropenia, lethargy, stiffness and generalised pulmonary changes, but was not caused by infectious agents; and pANCA were detected in the serum.

Cyclical episodes of rhinitis are rarely described in dogs. Whilst dogs with lymphoplasmacytic rhinitis can have periods of exacerbation, these are usually subtle in the initial stages. In more severely affected dogs changes in the severity of signs may be more obvious, but are usually associated with tapering treatment rather than being spontaneous (Mackin 2004). Dogs with cyclic neutropenia become infected with opportunistic pathogens during periods of neutropenia. These infections are typically associated with pyrexia and lethargy but are not usually limited to the respiratory tract (DiGiacomo et al. 1983; Niemeyer & Lothrop 2000). Platelet numbers may also cycle, so affected patients may have recurrent episodes of epistaxis, thus bearing some superficial resemblance to this case. Cyclic neutropenia is best described in grey collies, in which it is caused by an autosomal-recessive defect in neutrophil maturation, but isolated cases have been reported in other breeds (Alexander, Jones & Michel 1981; Latimer 1995). In this patient serial haematology results showed that neutropenia developed after the onset of clinical signs, excluding neutropenia as a trigger for the relapses, and platelet numbers remained normal.

More unusual cases of recurrent rhinitis with or without pneumonia develop when opportunistic pathogens (most commonly bacteria) are able to colonise the respiratory tract as a result of disturbed local or systemic immune defences. Examples include the rhinitis/bronchopneumonia syndrome of Irish wolfhounds (Clercx et al. 2003), primary ciliary dyskinesia (Norris 2004; Watson et al. 1999) and a syndrome associated with defective neutrophil function in young dobermans (Breitschwerdt et al. 1987). Respiratory signs of dogs affected with any of the above three conditions improve when they are treated with antibiotics, but then relapse once the antibiotics are discontinued. Although bacteria were cultured from this patient's nasal biopsies, treatment with antibiotics to which these pathogens were sensitive had not ameliorated clinical signs.

In dogs aspergillus rhinitis is the most common cause of severe turbinate destruction (Lefebvre, Kuehn & Wortinger 2005; Mathews 2004; Saunders et al. 2004). *Penicillium* is a rare differential diagnosis that causes identical clinical, imaging and rhinoscopic changes (Mathews 2004). Whilst fungal plaques may not be obvious on anterior rhinoscopy in all cases of aspergillus rhinitis (Johnson et al. 2006), this disease is not usually associated with systemic signs in dogs (Mathews 2004). If they do develop, pulmonary infiltrates most commonly appear nodular. In this case fungal rhinitis was excluded based on the absence of fungal plaques on rhinoscopy or histopathology of nasal biopsies, negative fungal cultures, atypical presentation and complete response to prednisone therapy.

Although nasal neoplasia, lymphoplasmacytic rhinitis, polyps and fungal rhinitides other than those caused by *Aspergillus* or *Penicillium* may cause turbinate destruction, they also cause mucosal inflammation and/or tissue proliferation, which results in increased soft-tissue opacities on CT and radiography (Holt & Goldschmidt 2011; Lefebvre et al. 2005; Wehner et al. 2008) and decreased nasal airflow on clinical examination. Again, they rarely cause systemic signs. They were not seriously considered in this case. The authors are not aware of any other causes of severe destructive rhinitis in dogs.

As the turbinate lysis was so dramatic, causes of atrophic rhinitis in other species were also investigated. Cats may develop severe turbinate lysis when infected by feline herpesvirus (Johnson et al. 2005). In pigs, atrophic rhinitis most commonly develops following infection with *Bordetella bronchiseptica* with or without toxigenic strains of *Pasteurella multocida* type D (Brockmeier & Register 2007). In humans it is associated with *Klebsialla ozenae* infection, sarcoidosis and GPA (DeShazo & Stringer 2011). Unlike aspergillus rhinitis, all these conditions are typically associated with systemic signs. In this case, bacterial culture did not identify any of the above bacteria and PCR detected no herpesvirus DNA in the biopsies. The clinical picture was quite different from sarcoidosis (Peckham & Spiteri 1996), but appeared similar to GPA (Pagnoux & Wolter 2012).

The nasal imaging changes were consistent with GPA (Mujagic et al. 2011). The ground-glass opacities noted on pulmonary CT were consistent with but not diagnostic of GPA, and are seen in approximately 30% of humans with GPA (Ananthakrishnan, Sharma & Kanne 2009). Pulmonary nodules (typically 2 cm – 4 cm in diameter, occasionally cavitating) are observed in 40% – 70% of affected people (Ananthakrishnan et al. 2009) but were not evident in this dog. A ground-glass appearance may be the result of pulmonary haemorrhage, infectious, inflammatory or neoplastic cellular infiltration, oedema or very early fibrosis (Agrawal et al. 2013; Dennler et al. 2011). In humans with GPA and in this dog it is most likely that the ground-glass pattern was the consequence of pulmonary haemorrhage or a small-vessel vasculitis. Lung biopsies would have been necessary to confirm the underlying pathology, but were not judged to be in the best interest of the patient.

Whilst immune-mediated diseases most commonly manifest in middle-aged female dogs, young animals may be affected: hypo-adrenocorticism is thought to result from immune-mediated destruction of the adrenals in most cases and often manifests in young adults (Feldman & Nelson 2004). Steroid responsive meningitis also typically affects adolescent or young adult dogs (Tipold & Schatzberg 2010). GPA is one of the most common primary systemic vasculitides diagnosed in children, with reported incidences of 0.03–3.2 cases per 100 000 children per year (Cabral et al. 2009).

Some immune-mediated diseases show spontaneous periods of exacerbation and remission, for example, steroid responsive meningitis-arteritis (Tipold & Schatzberg 2010) and immune-mediated neutropenia (Vargo, Taylor & Haines 2007; Weiss & Henson 2007). GPA is an idiopathic immune-mediated vasculitis, and affected humans show clinical signs similar to this dog's. As the presence of ANCA is very suggestive of this disease in humans, their presence was investigated in this case (Ozaki 2007; Pagnoux & Wolter 2012).

ANCA may be assayed by immunofluorescence or by the enzyme-linked immunosorbent assay (ELISA) (Ozaki 2007). Two immunofluorescence patterns are observed – cytoplasmic (cANCA) and perinuclear (pANCA). In humans the presence of cANCA appears highly correlated with detection of antibodies against proteinase 3 by ELISA, whilst the presence of pANCA is correlated with detection of antimyeloperoxidase antibodies on ELISA (Ozaki 2007; Santana et al. 2011). Whilst cANCA are highly specific for GPA and are present in at least 90% of affected people, pANCA are less specific and only detected in around 10% of humans with GPA (Ozaki 2007; Pagnoux & Wolter 2012; Santana et al. 2011). The only ANCA assay currently validated for use in dogs, to the authors’ knowledge, is the immunofluorescent pANCA assay set up by the Royal Veterinary College to study inflammatory bowel disease in dogs, but the antigen the anti-dog IgG antibodies bind to has not been identified in dogs (Allenspach et al. 2004).

In humans pANCAs are increased in a variety of immune-mediated diseases, including rheumatoid arthritis, auto-immune hepatitis, inflammatory bowel disease (ulcerative colitis and Cohn's disease), systemic lupus erythematosis, glomerulonephritis, primary systemic vasculitides including GPA, as well as in a variety of tumours (Karagianni et al. 2012; Mancho et al. 2011; Mancho et al. 2010). pANCAs were also detected in some humans infected with *Mycobacterium*,* Bartonella* or *Leishmania* (Karagianni et al. 2012). Authors speculated that the high prevalence of pANCA seroreactivity associated with some infectious or neoplastic diseases develops because the primary disease causes a vasculitis or other immune dysregulation (Karagianni et al. 2012; Mancho et al. 2011).

pANCAs have been shown to be increased in dogs with diet-responsive or idiopathic inflammatory bowel disease (Luckschander et al. 2006; Mancho et al. 2011), soft-coated Wheaten terriers with protein-losing enteropathy and/or nephropathy (Allenspach et al. 2008; Wieland et al. 2012), primary immune-mediated haemolytic anaemia and a variety of vector-borne diseases (*Bartonella*,* Borrelia burgdorferi*,* Ehrlichia* and **Leishmania*)* (Karagianni et al. 2012) as well as intestinal lymphoma (Mancho et al. 2011). The infections listed are all associated with both immune-mediated disease and vasculitis in dogs (Baneth & Solano-Gallego 2012; Breitschwerdt & Chomel 2012; Greene, Straubinger & Levy 2012; Reardon & Pierce 1981), but were excluded in this dog based on the signalment, clinical signs, diagnostic testing and treatment response.

Humans with GPA typically require long-term immunosuppressive treatment. Most people treated with steroids alone have either progressive signs or severe steroid side-effects that are fatal within–12 months of diagnosis (Langford 2011; Santana et al. 2011). Remission rates and long-term survival are markedly improved when cyclophosphamide is added to the treatment (Langford 2011; Santana et al. 2011). This dog was not treated with cyclophosphamide from the outset because this treatment would have required more intensive monitoring and the dog lived more than an hour's drive away from the primary author. In addition, there were no previous reports of this disease in dogs to indicate that it was essential. It would have been added had she not responded to monotherapy.

In this case the increased pANCA, cyclical nature of the disease, exclusion of known infectious diseases that could cause similar signs, complete response to prednisone monotherapy and long disease-free follow-up offer strong evidence that this dog had primary immune-mediated disease.

The evidence that this immune-mediated disease was indeed GPA is less definitive. As discussed above, diagnosis of GPA is based on an algorithm in humans and can only be made once infectious and neoplastic diseases have been excluded (Watts et al. 2007) (much like systemic lupus erythematosis is in dogs). A variety of algorithms are reported, suggesting none is perfect. They typically include several clinical, histopathological and serological criteria. This patient had an erosive rhinitis, ground-glass opacities on pulmonary CT, systemic signs (pyrexia, lethargy) and was positive for pANCA. Based on the criteria of a 2007 review (Ozaki 2007), this patient probably had GPA. Based on the criteria of Watts and others as well as those of the American College of Rheumatology, a firm diagnosis of GPA would have been made in this patient had she been human (Leavitt et al. 1990; Watts et al. 2007).

Diagnostic algorithms developed for one species should not be transferred to another without some evidence to confirm that they are reliable. A literature search yielded a single case report describing a dog with histopathological lesions resembling GPA (Krug et al. 2006). This dog presented with severe proliferative gingivitis only. A case series of 176 dogs presenting with epistaxis identified a single case associated with vasculitis and moderate thrombocytopenia. Paired serum titres failed to reveal infection with *E. canis*, *Borrelia burgdorferi* or *Rickettsia* sp., but clinical signs resolved following treatment with enrofloxacin and doxycycline, so this is unlikely to have been GPA (Bissett et al. 2007). Thus there are no prior confirmed cases of GPA causing rhinitis in dogs and diagnostic algorithms have thus not been investigated for this disease in this species. For a firm diagnosis of GPA to have been made in this dog, cANCA or typical histopathological changes would have to have been found in addition to the changes listed above.

Other idiopathic ANCA-positive small-vessel vasculitides reported in humans include microscopic polyangiitis (MPA) and eosinophilic granulomatosis with polyangiitis (EGPA or Churg-Strauss syndrome). Whilst humans with MPA are much more likely to have circulating pANCA than cANCA, less than 30% present with a rhinitis and the rhinitis is not erosive. Glomerulonephritis is also present at diagnosis in the majority of cases (Chung & Seo 2010; Pagnoux & Wolter 2012). EGPA was not seriously considered as there was no eosinophilic infiltrate on the biopsies.

## Conclusion

This is the first report on the use of pANCA to investigate rhinitis in dogs. It is also, to the authors’ knowledge, the first description of a relapsing haemopurulent lytic rhinitis in this species. The concurrent manifestations of erosive haemopurulent rhinitis, ground-glass opacities on pulmonary CT, pyrexia and listlessness resemble GPA as described in humans. Clinical signs resolved promptly and completely once immunosuppressive doses of prednisone were administered.
